# Palatal changes after treatment of functional posterior cross-bite using elastodontic appliances: a 3D imaging study using deviation analysis and surface-to-surface matching technique

**DOI:** 10.1186/s12903-023-02731-7

**Published:** 2023-02-02

**Authors:** Antonino Lo Giudice, Vincenzo Ronsivalle, Cristina Conforte, Giuseppe Marzo, Alessandra Lucchese, Rosalia Leonardi, Gaetano Isola

**Affiliations:** 1 Department of General Surgery and Medical-Surgical Specialties, Section of Orthodontics, University of Catania, Catania, Italy; 2 Department of General Surgery and Medical-Surgical Specialties, School of Dentistry, University of Catania, Catania, Italy; 3 Department of Health, Life and Environmental Science, University of L’Aquila, Piazza Salvatore Tommasi, 67100 L’Aquila, Italy; 4 Department of Dentistry, Dental School, IRCCS San Raffaele Hospital, Vita-Salute San Raffaele University, Milan, Italy; 5 Policlinico Universitario “Gaspare Rodolico - San Marco”, Via Santa Sofia 78, 95123 Catania, Italy

**Keywords:** Orthodontics, Digital orthodontics, Digital dentistry, Elastodontics, Crossbite

## Abstract

**Background:**

The present study aimed to evaluate the changes in palate dimension and morphology after treatment of functional posterior crossbite (FPXB) with elastodontic devices (EAs).

**Methods:**

The treatment group (TG) consisted of 25 subjects (mean age 7.3 ± 0.9 years) who received treatment with EA for one year. The control group (CG) comprised 14 untreated subjects (mean age 6.8 ± 0.7 years). Inclusion criteria: intra-oral scan registered before (T0) and after treatment (T1), FPXB with a mandibular shift towards the crossbite site of ≥ 2 mm, class I molar relationship. Exclusion criteria: missing teeth, anterior crossbite, temporomandibular disorders, previous orthodontic treatment, carious lesions, mobility of deciduous posterior teeth, craniofacial deformities. Digital models were analyzed to assess the inter-canine (ICW) and inter-molar widths (IMW) and the corresponding emi-lateral measurements (eICW and eIMW) using the median palatine plane as reference. According to a specific 3D imaging technology, the morphology and symmetry of the palate was investigated by analysing the 3D deviation between the two specular models of the palate.

**Results:**

At T0, both groups showed a significantly narrower dimension of eICW and eIMW at the crossbite side compared to the non-crossbite side (*p* < 0.05). Also, the 3D deviation analysis demonstrates a limited matching percentage of the original/mirrored models in both TG (81.12%) and CG (79.36%), confirming the asymmetry of the palate. The area of mis-matching was located at the alveolar bone level. At T1, subjects in the TG showed a significant increment of ICW and IMW (*p* < 0.05), a reduction of the differences of eICW and eIMW between both sides (*p* < 0.05) and an increment of the percentage matching (TG = 92.32%) (*p* < 0.05), suggesting a significant recovery of the palatal asymmetry. No significant changes were found between T0 and T1 in the CG (*p* > 0.05).

**Conclusions:**

EAs could be successfully used to correct FPXB in mixed dentition and could restore the harmonious development of the palate in children.

## Background

Orthodontic therapy at the growing stage consists of a preventive approach for treating malocclusions that are not self-correcting with age [1–3]. Nevertheless, general consent for interceptive treatment has not been defined since some studies suggest that early treatment could lead to a stable occlusion [4, 5], while other studies indicate that children would not benefit from early intervention, except for a transitory increase in self-esteem [6, 7]. Early treatment is advocated when it is crucial to eliminate factors affecting the harmonious development of dental arches, often resulting in compensatory skeletal and dentoalveolar adaptation to maintain a stable function and occlusion [8].

Posterior cross-bite is a type of malocclusion with remarkable prevalence (7–23%) in deciduous and mixed dentition [9] and it is often associated with maxillary hypoplasia or transverse dentoalveolar contraction [10]. It can occur both unilaterally or bilaterally. When unilateral, posterior cross-bite is often caused by a functional shift of the mandible towards the cross-bite due to a mild bilateral maxillary constriction which generates occlusal interference leading to a functional shift of the mandible towards the cross-bite in centric occlusion. From a clinical perspective, functional posterior crossbite (FPXB) requires early treatment to prevent the asymmetric pattern of mandibular growth [11]. Skeletal maxillary expansion or dentoalveolar expansion with or without occlusal grinding of deciduous dentition represents the treatment approach for cross-bite correction at the growing stage [11, 12].

Elastodontics is an interceptive therapy that uses removable appliances made of silicone elastomer, which allows the development of light, biological elastic forces. These forces would enable the correction of malocclusions at developmental age, correcting the position of teeth and potentially affecting growth [13–15]. Elastodontic appliances (EAs) originate from its precursor Occlus-O-Guide®, or Eruption Guidance Appliance (EGA), designed to generate minor positional correction or to guide dental eruption. The elastomeric material promotes the orthodontic movement in synergy with the neuromyofascial system, while the vestibular flanges prevent the perioral muscles from influencing the movement of the teeth [16]. Several types of EAs are available in the market and designed for the early treatment of different forms of malocclusion. In particular, there are EAs recommended for the expansion of the maxillary arch, avoiding the necessity for orthopedic treatment (maxillary skeletal expansion) in those subjects affected by mild constriction of the maxillary arch. Although EAs are widely used among orthodontists and pedodontists, the scientific evidence regarding their effectiveness is scarce and limited to the early treatment of crowding, overbite, overjet and molar relationships [2, 17–19]. To date, there is no evidence regarding the efficacy of EAs in treating mild maxillary contraction and FPXB in children.

In this regard, the present study aimed to evaluate the changes in the maxillary arch and morphology of the palate after treatment of FPXB in a retrospective cohort of subjects in early mixed dentition. For this purpose, we used a specific 3D imaging technology involving superimposition of pre-treatment and post-treatment maxillary intra-oral scans to evaluate the morphological changes of the palate during the treatment stages.

## Methods

### Study sample

This study was carried out following the Helsinki Declaration on medical protocols and ethics and was approved by the Local Institutional Review Board. Informed consent for orthodontic treatment and for research purposes was signed by parents of all the included subjects. The sample of this retrospective study included 39 subjects (mean age 7 ± 0.8 years) seeking orthodontic treatment at the Department of Orthodontics of the University of Catania, between September 2017 and December 2020. Inclusion criteria were: (1) intra-oral scan registered before treatment (T0) and after appliance removal (T1), (2) posterior cross-bite of at least two maxillary posterior teeth, (3) mandibular shift towards the cross-bite site of ≥ 2 mm in centric occlusion and not in centric occlusion (FPXB), (4) class I or edge-to-edge molar relationship. Exclusion criteria: missing teeth, anterior cross-bite, temporomandibular disorders, previous orthodontic treatment, carious lesions, mobility of deciduous posterior teeth, craniofacial deformities.

The treatment group (TG) consisted of 25 subjects (mean age 7.3 ± 0.9 years) who received the AMCOP Integral/Basic activator (Ortho Protec, BA, Italy). In comparison, the control group (CG) consisted of 14 subjects (mean age 6.8 ± 0.7 years) who postponed the orthodontic treatment one year after the orthodontic consultation due to the social/health restrictions caused by the COVID-19 pandemic.

### Treatment

**Fig. 1:**
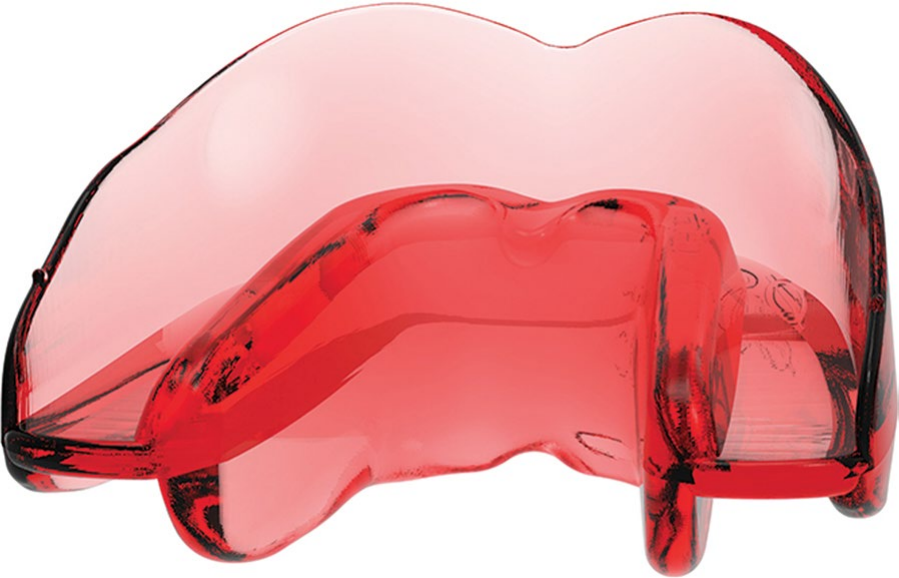
Integral/Basic AMCOP SC bio-activator (Ortho Protec, BA, Italy)

The AMCOP Integral/Basic activator is an elastomeric preformed device designed to favor the expansion of both arches; it features oral flanges that reduce the interference of perioral muscles and allow patients to place the tongue in the correct posture at the palatine spot. Figure 1 illustrates the EA used in this study. The fitting size was selected considering the inter-molar diameter and the inclination of the incisors for each patient. All participants in the TG were recommended to wear the appliance at night and for two hours during the day. The patients were also instructed to bite the device during daily wear, keeping the lips in contact. Once the correction of FPXB was achieved, patients were instructed to wear the appliance only two hours per day as retention. Patients in the treatment group may require slight occlusal grinding of maxillary deciduous canines to facilitate the elimination of occlusal interferences. Intra-oral scans and bite registration were taken before treatment (T0) and after 12 months (T1) (Carestream 3600, Carestream Dental LLC, Atlanta, GA, USA).

### Measurements

All digital models were imported into Ortho Analyzer software (3Shape A/S, Copenhagen, Denmark) to perform linear measurements and 3D assessment of the morphology of the palate both at T0 and T1.

**Fig. 2:**
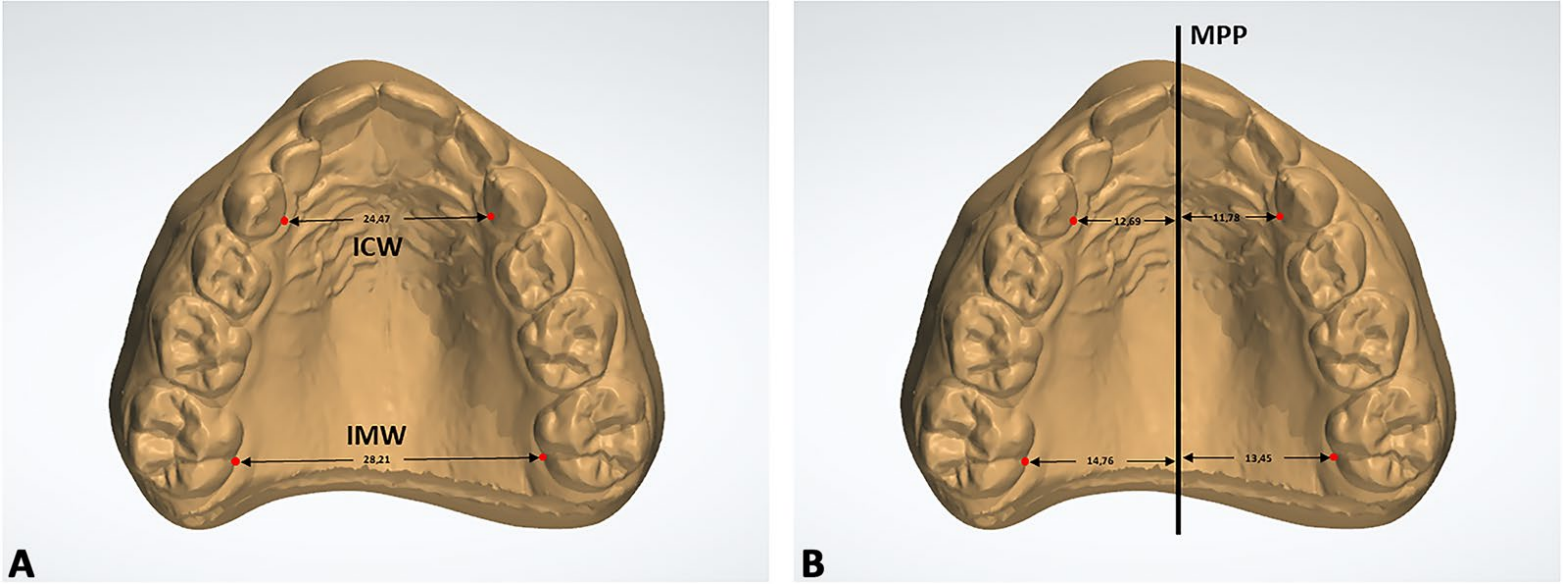
Linear measurements performed in this study to assess transverse dimension of the palate. **A** inter-canine width (ICW), inter-molar width (IMW); **B** emi-intercanine width (eICW), emi-intermolar width (eIMW). The median palatal plane (MPP) was drawn through two landmarks detected along the median palatal raphe and showed in red. The first landmark identified the point on the median palatal raphe adjacent to the second ruga. The second landmark was placed on the median palatal raphe 1 cm distal to the first point. eICW and eIMW represent respectively the linear distance from the midpoint of the dento-gingival junction of the primary canine and first molar to the MPP

Firstly, the transverse dimension of the palate was calculated at the level of permanent first molars (IMW, intermolar width) and deciduous canines (ICW, intercanine width), using the landmark placed at the center of the dento-gingival junction (Fig. 2a) as reference. The median palatal plane (MPP) was traced on digital casts using two landmarks identified along the median palatal raphe, respectively the point (1) on the median palatal raphe adjacent to the second ruga and the point (2) on the median palatal raphe 1 cm distal to point 1 [8]. Once the MPP was traced, the following measurements were performed (Fig. 2b):

- eICW (emi-intercanine width): the distance between the midpoint at the dento-gingival junction of the primary canine from the crossbite and non-crossbite sides compared with the MPP
- eIMW (emi-intermolar width): the distance between the midpoint of the dento-gingival junction of the first molar from the crossbite and non-crossbite sides compared with the MPP.

**Fig. 3:**
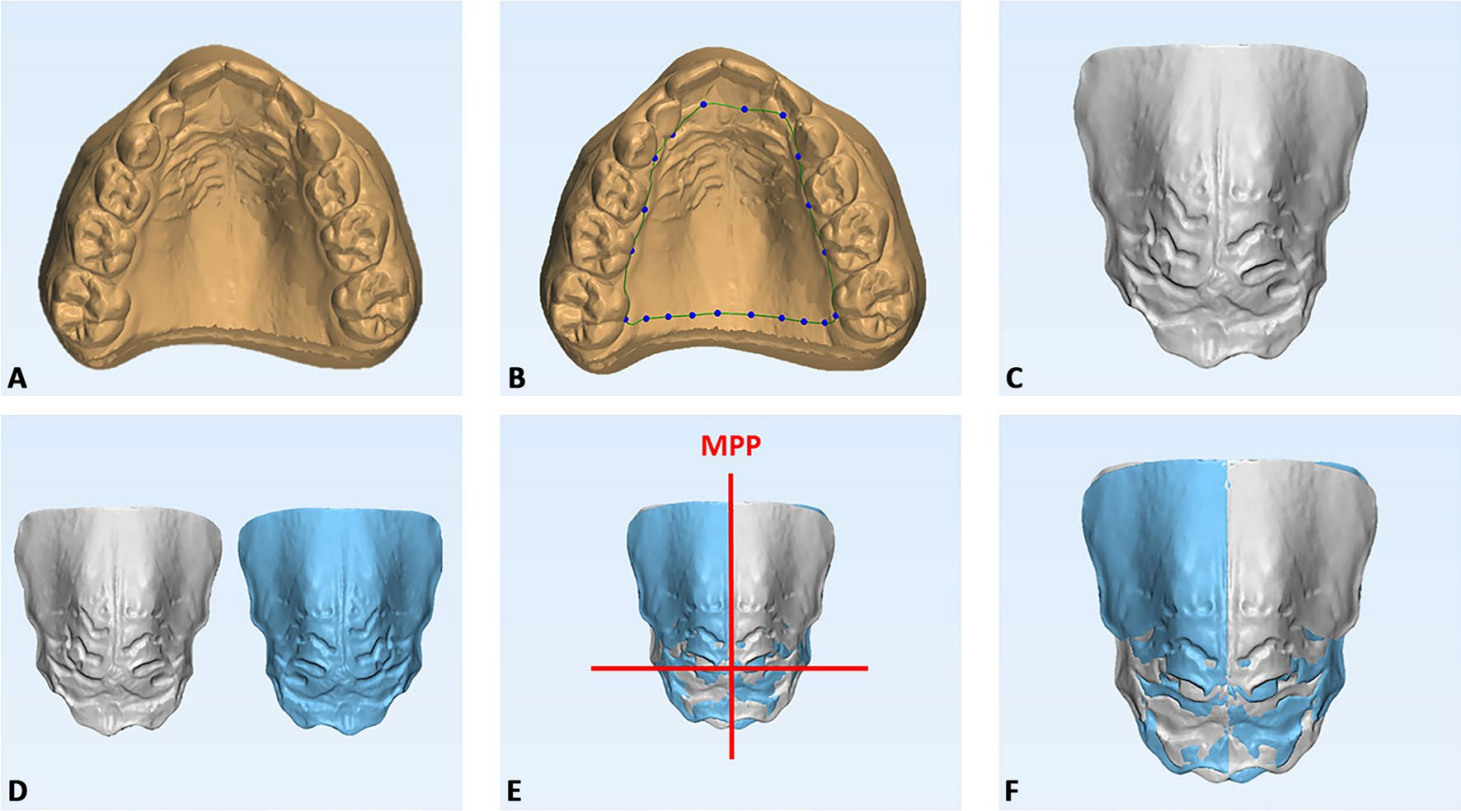
Digital workflow for the analysis of maxillary models from intraoral scans. **A**, **B** Segmentation of the palate by connecting the most apical points of the lingual dento-gingival junctions of all teeth; **C** Generation of the 3D model of the palatal vault; **D** Generation of duplicated mirrored model of the palatal vault; **E** Superimposition of the original and mirrored models using the MPP plane and its perpendicular plane as reference; **F** Final superimposition adjustment using ‘best-fit’ alignment algorithm

To verify the morphological changes (symmetry/asymmetry) and perform surface analysis of the palate, a specific 3D imaging technology involving superimposition of T0 and T1 intra-oral scans was carried out, according to a consolidated methodology [8]. The procedure involved four steps:

*Segmentation of the palate* (Ortho Analyzer software, 3Shape A/S, Copenhagen, Denmark): a 3D model was defined by generating a gingival plane passing through all the most apical points of the dento-gingival junction of all teeth, from the right 1st molar to the left 1st molar (Fig. 3a–c).

*Mirroring* (Geomagic Control™ X (version 2017.0.0, ‘3D Systems’, Rock Hill, USA): a duplicated mirrored model of the palate was generated for both T0 (model T0m) and T1 (model T1m) stages using the midpalatal plane (MPP) as reference, that is the line passing through a point placed at the level of the second rugae and a second point 1 cm distal, along the palatal raphe (Fig. 3d–e).

*Surface registration*: the original and mirrored palate models were superimposed via preliminary registration using MPP as the reference plane. Final registration was executed using the “Best-fit alignment” feature in the Geomagic Control X software (Fig. 3f).

**Fig. 4:**
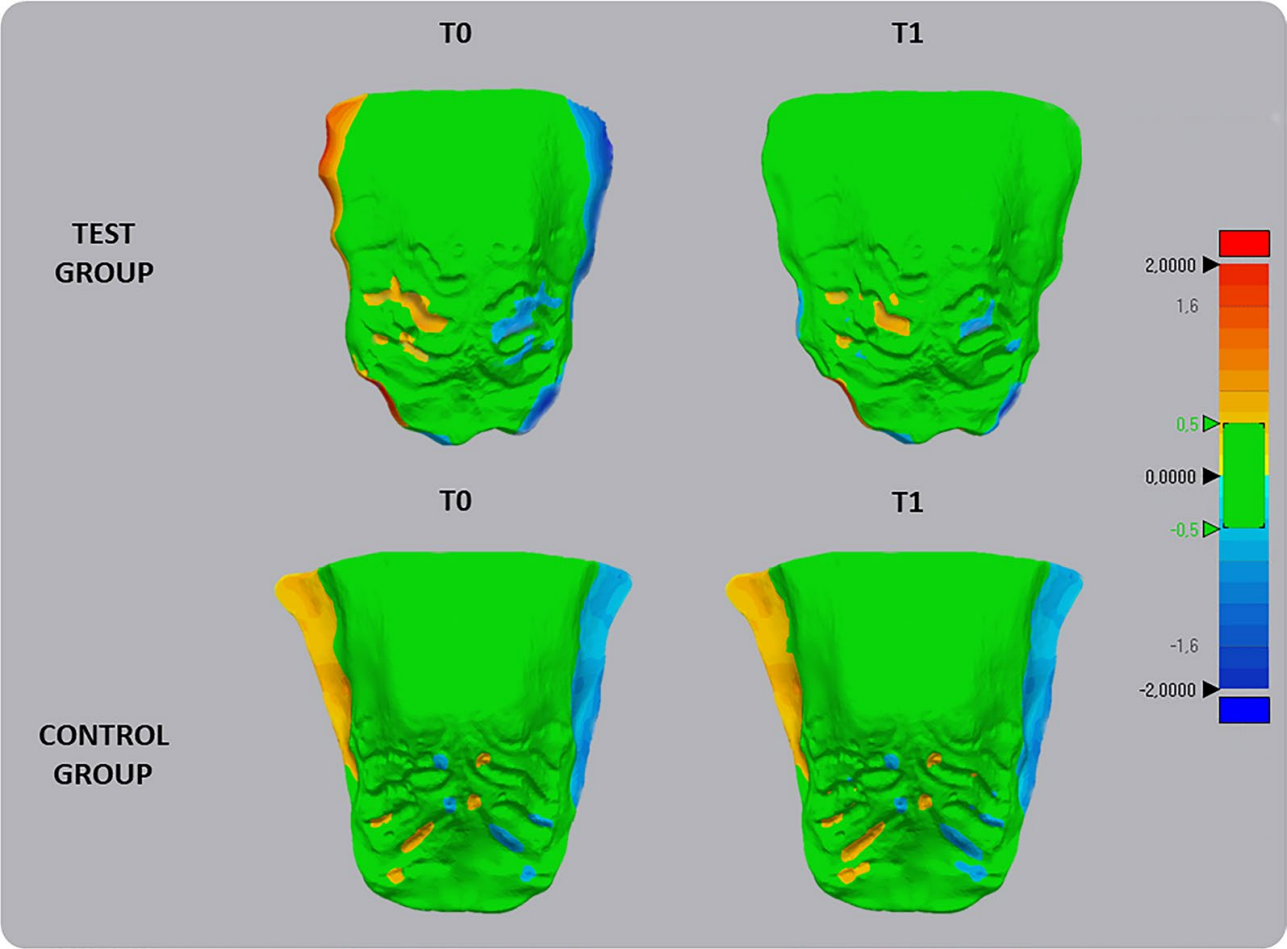
Deviation analysis and calculation of the percentage of matching between the original and speculated models of the palate detected at T0 and T1 in both test group and control group. The RGB coloured scale bar (millimetres) is shown on the right: the upper (red) and lower (blue) parts of the scale indicate the maximum positive and negative deviations. Green indicates the tolerance range, set to 0.3 mm

*Deviation analysis and matching percentage calculation*: using the same software, the deviation analysis automatically calculated the mean and maximum values of the linear distances (Euclidean distance) between the surfaces of the two palatal models, measured across 100% of the surface points. The analysis was complemented by the visualization of the 3D color-coded maps, set at 0.5 mm range of tolerance (green colour), to evaluate and locate the discrepancy between the model surfaces (Fig. 4). The percentages of all the distance values within the tolerance range were calculated. These values represented the degree of correspondence between the original and the mirrored models and, therefore, provided quantitative data of the morphological characteristics of the palate detected at T0 and T1.

The entire workflow, including segmentation and relative generation of the mask, was carried out by the same experienced operator with 15 years of experience in clinical and digital orthodontics (A.L.G.). The same operator performed measurements four weeks later to obtain data for intra-operator reliability. A second expert operator also performed the digital workflow to obtain data to assess inter-operator reliability (V.R.).

### Statistical analysis

The sample size calculation was performed using data of a previous study [8]. The analysis showed that 13 subjects were required for each treatment group to detect a mean difference of 1.52 mm in the eIMW measurements between cross-bite side and non-crossbite side in subjects affected by FPXB in mixed dentition, with a power of 80% and a significance level of 0.05.

Descriptive statistics were carried out to analyze the demographic and clinical characteristics of the TG and CG groups. The Student’s *t*-test and chi-square test compared numerical (age) and categorical (gender, skeletal maturity) characteristics between TG and CG.

Preliminary data analysis was performed using the Shapiro–Wilk test and Levene’s test, respectively, to assess data distribution and equality of variance. Since data showed normal data distribution, parametric tests were used. Paired Student’s *t*-test was used for inter-timing comparison of bilateral linear measurements and 3D data and an unpaired Student’s *t*-test was used for inter-groups comparison of the same measurements. Concerning emi-lateral measurements, unpaired Student’s *t* test was used to compare cross-bite side and non-cross-bite side (intra-timing assessment) and to compare the mean difference of the same data between the two groups (inter-groups mean difference evaluation). Instead, paired Student’s *t*-test was used for the inter-timing assessment of the mean difference between cross-bite and non-cross-bite side. Statistical significance was set at *p* < 0.05. Intra-examiner reliability was assessed using the Intraclass Correlation Coefficient (ICC). Datasets were analyzed using SPSS® version 24 Statistics software (IBM Corporation, 1 New Orchard Road, Armonk, New York, USA).

## Results

The descriptive statistics of the TG and CG are reported in Table 1. In the TG, a statistically significant increment of IMW and ICW was registered between T0 and T1 (*p* < 0.05); on the contrary, a slight reduction of the same measurements was recorded in the CG, although these findings were not supported by statistical significance (*p* > 0.05) (Table 2).

**Table 1:** Demography and clinical characteristics of the sample of the study

Sample characteristics	Total (n = 39)	Total (n = 25)	Total (n = 14)	Significance
	Mean/n	Mean/n	Mean/n	
Mean age	7 (± 0.8)	7.3 (± 0.9)	6.8 (± 0.7)	NS
*Gender*				
Male	16	10	6	NS
Female	23	15	8	
*Skeletal maturity*				
CVMS 1	36	23	13	*p* < 0.05
CVMS 2	3	2	1	

*p*-value for comparison of group means by *t*-test or differences in proportion calculated by chi-square test

CVMS, cervical vertebrae maturation stages

**Table 2:** Comparative assessment of bilateral palatal linear measurements recorded in the treated group (TG) and control group (CG)

	ICW		*p* value*	T1–T0	*p* value**	IMW		*p* value*	T1–T0	*p* value**
	T0	T1				T0	T1			
TG group	24.2 (± 1.35)	26.4 (± 1.67)	*p* < 0.05	2.2 (± 0.4)	*p* < 0.05	28.63 (± 1.63)	31.2 (± 1.81)	*p* < 0.05	2.9 (± 0.7)	*p* < 0.05
CG group	25.03 (± 1.40)	24.94 (± 1.51)	NS	0.09 (± 0.17)		29.62 (± 2.03)	29.46 (± 2.16)	NS	0.16 (± 0.24)	

ICW, distance between the dento-gingival junctions of primary canines; IMW, distance between the dento-gingival junctions of first molars

*p values based on paired Student’ *t* test and set at *p* < 0.05 (inter-timing comparison)

**p values based on Independent Student’ *t* test and set at *p* < 0.05 (inter-groups comparison); NS, non significant

At T0, both eICW and eIMW were significantly smaller on the cross-bite side compared to the non-cross-bite side in both groups (*p* > 0.05), suggesting a mild asymmetry of the maxillary arch. The same findings were recorded at T1; however, the eICW and eIMW mean differences between both sides were significantly reduced compared to T0 (*p* < 0.05) in the TG, while they remained similar in the CG (*p* > 0.05) (Table 3). These findings would suggest that the expansion was greater at the cross-bite side, reducing the asymmetry of the maxillary arch even in comparison with the CG (*p* < 0.05) (Table 3).

**Table 3:** Comparative assessment of emilateral palatal linear measurements recorded in the treated group (TG) and control group (CG)

			eICW	Mean diff	*p* value*	*p* value**	*p* value**			eIMW	Mean diff	*p* value*	*p* value**	*p* value**
TG	T0	CBS	11.67 (± 1.32)	0.86 (± 0.15)	*p* < 0.05	*p* < 0.05	*p* < 0.05	T0	CBS	13.53 (± 1.19)	1.54 (± 0.21)	*p* < 0.05	*p* < 0.05	*p* < 0.05
		nCBS	12.53 (± 1.28)						nCBS	15.07 (± 1.22)				
	T1	CBS	12.98 (± 1.40)	0.44 (± 0.16)	NS			T1	CBS	15.15 (± 1.27)	0.88 (± 0.19)	*p* < 0.05		
		nCBS	13.42 (± 1.37)						nCBS	16.03 (± 1.28)				
CG	T0	CBS	13.01 (± 1.21)	0.99 (± 0.10)	*p* < 0.05	NS		T0	CBS	14.06 (± 1.41)	1.50 (± 0.15)	*p* < 0.05	NS	
		nCBS	12.02 (± 1.24)						nCBS	15.56 (± 1.46)				
	T1	CBS	12.96 (± 1.35)	0.98 (± 0.10)	*p* < 0.05			T1	CBS	13.95 (± 1.51)	1.54 (± 0.14)	*p* < 0.05		
		nCBS	11.97 (± 1.39)						nCBS	15.49 (± 1.50)				

eICW, distance between mild palate plane and the centre of dento-gingival junctions of primary canine; eIMW, distance between mild palate plane and the centre of dento-gingival junctions of first molar; CBS, cross-bite side; nCBS, non cross-bite side

*P values based on Independent Student’ *t* test for intra-timing side-to-side measurements and set at *p* < 0.05

**P values based on paired Student’ *t* test for inter-timing mean differences and set at *p* < 0.05; NS, non significant

***P values based on Independent Student’ *t* test for inter-groups comparison of the mean of differences and set at *p* < 0.05

Results of the intra-timing analysis at T0 (T0 vs T0m) showed a limited percentage agreement, respectively of 81.12% (± 3.03) in TG and 79.36 (± 2.95) in the CG, suggesting a slight morphological asymmetry of the maxillary anatomy. At T1, there was a statistically significant increment of the percentage of agreement of palatal surfaces (T1 vs T1m = 92.32% ± 4.05) in the TG (*p* < 0.05); instead, in the CG, there was a slight reduction of the palate matching percentage (T1 vs T1m = 77.47% ± 3.14), however without statistical significance (*p* > 0.05) (Table 4). Concerning deviation analysis, the palatal vault showed a prevalence of green color, indicating that this area coincided with the original and the mirrored models. Instead, the color-coded map showed an intense blue color on one side of the palatal surface of the alveolar process and an intense red color on the other. This data would suggest that the palatal asymmetry was mainly confined to the lower part of the palate at the alveolar processes level, as showed in Fig. 4.

**Table 4:** Comparison of intra-timing matching percentage agreement between original and mirrored palate models in the study and control groups

		Matching%	SD	*p* value*	Mean diff	SD	*p* value**
TG	Mirroring T0	81.12	3.03	*p* < 0.05	11.2	1.86	*p* < 0.05
	Mirroring T1	92.32	4.05				
CG	Mirroring T0	79.36	2.95	NS	− 1.89	0.27	
	Mirroring T1	77.47	3.14				

*p value set at *p* < 0.05 based on paired Student’s *t* test for intertiming comparisons

**p value based on Independent Student’s *t* test for inter-groups comparisons

No differences were found between intra-operator readings, with excellent correlation indexes ranging from 0.924 to 0.936 for linear measurements and from 0.898 to 0.917 for 3D analysis. Similarly, no differences were found between intra-operator readings, with excellent correlation indexes ranging from 0.901 to 0.920 for linear measurements and from 0.887 to 0.931 for 3D analysis.

## Discussion

EAs are widely used for interceptive orthodontic treatment in mixed dentition, and FPXB is one of the most frequent malocclusions occurring during this stage [11]. The available scientific evidence concerning the effects of EAs is scarce and limited to the early treatment of anterior crowding and sagittal and vertical inter-arches discrepancies [17–19]. To the best of our knowledge, this is the first study in the literature that investigated the effects of EAs in treating FPXB in a cohort of growing subjects. In particular, we analyzed the changes in the maxillary arch and the palate morphology since the successful treatment of FPXB is based on the transverse expansion of the upper jaw. For this purpose, we used a specific 3D imaging technology that allowed the analysis of the symmetry of the palate vault to obtain a comprehensive evaluation of the morphological characteristics of the subjects affected by FPXB and the potential morphological changes that occurred due to treatment.

The present study included a control group of untreated subjects to discriminate the changes related to the treatment from those that occurred due to growth. The control group comprised subjects who did not start the orthodontic treatment due to the social/health restrictions caused by the COVID-19 pandemic. The same children had the opportunity to receive the treatment one year later, avoiding the ethical issues related to the recruitment of growing subjects in the control group for the scientific purpose [20].

The results of the present study demonstrated a mild asymmetric maxillary arch and asymmetric morphology of the palate in subjects presenting FPXB. In fact, in both TG and CG groups, the eICW and eIMW were narrower on the cross-bite side compared to non cross-bite side (almost 1 mm for eICW and about 1.5 mm for eIMW), and these results were in agreement with previous findings [21]. We also found a limited percentage of agreement obtained by overlapping the original palate model with the mirrored model, corresponding to 81.12% in TG and 79.36% in the CG. These data would suggest that the asymmetry was not limited to the dentition (inter-canine and inter-molar widths) but also involved the skeletal components. According to the deviation analysis, the mismatching between original and mirrored models was detected in the palate region proximate to the dentoalveolar processes, without the interest of the basal bone. This pattern of asymmetry involving the alveolar process has been documented in the presence of FPXB, and has been explained as the adaptive bending of the maxillary alveolar process of the cross-bite side for maintaining occlusal contacts with the antagonist mandibular dentition due to mandibular shift [8, 22].

All subjects in the treated group showed complete correction of the FPXB with the coincidence of the upper and lower midlines in both centric occlusion and centric relation. Subjects in the TG showed a significant increment of both IMW and ICW, suggesting that the EA used in this study effectively increased the transverse dimension of the maxillary arch. Interestingly, we found that the increment of IMW and ICW was greater on the cross-bite side compared to the non-cross-bite side, with a significant reduction of the mean difference between both sides at T1. The same occurred at the skeletal level since the values of matching percentage increased from 81.12% at T0 to 92.32% at T1. As a consequence, subjects in the TG showed a significant improvement in maxillary asymmetry after one year of therapy. Instead, no differences were found between measurements taken at T0 and T1 in the CG; thus, the changes found in the TG could not be related to growth, instead they were likely favored by the interceptive treatment of FPXB.

In this regard, the design of EA used in this study features flanges that isolate the dentoalveolar processes and the palate from the surrounding structures, particularly the perioral muscles that act asymmetrically in the presence of unilateral posterior cross-bite [11, 23]. Thus, it is possible that EAs, by rebalancing the pe-rioral musculatures, could somehow favor re-establishing a normal pattern of development of the palate and alveolar processes by contrasting the forces that may interfere during its growth. This effect is that advocated with the usage of functional appliances that isolate perioral muscles such as Frankel device [24], although with different design, functional properties, and clinical applications. EAs also act as a myofunctional regulator that actively rebalance muscle forces [25]. Further studies are encouraged to elucidate this aspect, including comparative assessments using different types of appliances.

Subjects in the CG did not show spontaneous correction of the FPXB, confirming that posterior cross-bite is not self-correcting and requires early treatment to prevent the malocclusion from being perpetuated in permanent dentition [12]. Also, considering the adaptive bending of the maxillary alveolar processes (asymmetry) found in this study and previous investigations [8, 21], it may be postulated that the asymmetry of the palate can worsen with age, complicating the biomechanics of expansion at a later stage. In this regard, the main goal of the early treatment of FPXB is to eliminate occlusal interference as an opportunity to restore form and function [26].

Subjects in the TG were affected by mild bilateral maxillary contraction and required dento-alveolar expansion with or without selective adjustment and occlusal guiding as early intervention, as corroborated by previous evidence [12, 27]. Skeletal maxillary expansion or dentoalveolar expansion with occlusal grinding of deciduous dentition represents the treatment approach for cross-bite correction at the growing stage [11]. Since treatment of FPXB generally requires the expansion of maxillary arch by using skeletal or dentoalveolar expanders, depending on the area and severity of the deficiency, a future area of investigation could be the assessment of palatal changes occurred with different appliances used for the treatment of FPXB in mixed dentition. Furthermore, further studies should be performed to confirm and evaluate the long-term stability of the palatal improvements with elastodontic treatment.

### Limitations

- The small sample size, in particular, the limited control group is one of the major concerns of the present investigation. However, considering the general ethical restriction related to the recruitment of control groups for scientific purposes, it represents a heritage sample of untreated subjects that could be used for further comparative investigations.
- Both treatment and control groups were retrospectively recruited. Thus, it was impossible to control a priori potential confounding factors or specific variables that may have affected data outcomes. However, according to the inclusion/exclusion criteria, we were able to analyze a homogeneous study sample concerning mean age, dental and skeletal maturation stage. Future randomized studies are warmly encouraged to overcome the reported limitation of study design.

## Conclusions

- An asymmetric pattern of palatal growth was found, mostly located at the dento-alveolar process, which significantly improved in the tested group compared to controls.
- EAs could be successfully used for the correction of FPXB associated with mild maxillary contraction mitigate and could contribute to restore the harmonious development of the palate.

## Data Availability

The datasets used and analysed during the current study are available from the corresponding author on reasonable request.

## Abbreviations

EAs: Elastodontic appliances
EGA: Eruption guidance appliance
FPXB: Functional posterior crossbite
IMW: Intermolar width
ICW: Intercanine width
MPP: Median palatal plane
eICW: Emi-intercanine width
eIMW: Emi-intermolar width
